# *Staphylococcus aureus* Toxins: Armaments for a Significant Pathogen

**DOI:** 10.3390/toxins11080457

**Published:** 2019-08-03

**Authors:** William R. Schwan

**Affiliations:** Department of Microbiology, University of Wisconsin-La Crosse, 1725 State St., La Crosse, WI 54601, USA; wschwan@uwlax.edu

*Staphylococcus* species are common inhabitants of humans and other animals. *Staphylococcus aureus* is responsible for hundreds of thousands of skin and soft tissue infections [1] and is a significant cause of bloodstream infections in humans [2]. Several different exotoxins are produced by *S. aureus*, including enterotoxins, hemolysins, and leukocidins that kill white blood cells [3,4]. These exotoxins lead to direct damage to the host, modulate immune defenses, and may have an indirect effect on the host by mounting a cytokine storm. Early studies on staphylococcal exotoxins include work by Bayliss who identified that *S. aureus* enterotoxins caused vomiting [5], Marks investigation of hemolysins [6], and Blobel et al. demonstrating the lethal properties of leukocidins on human white blood cells [7]. When community-associated methicillin-resistant (CA-MRSA) *S. aureus* emerged, sequencing of the first CA-MRSA strain showed the acquisition of new pathogenicity islands harboring new exotoxin genes that contribute to human disease [8]. Thus, *S. aureus* continues to evolve in part by acquiring new exotoxin genes.

This special issue is focused on staphylococcal toxins and the impact they have on mammalian health. Several studies examined the role toxins play in *S. aureus* pathogenesis, whereas other papers explored new therapeutics aimed at limiting the action of the toxins. A couple of review papers give good overviews as to why toxins are crucial for specific aspects of *S. aureus* infections.

A paper by Bretl et al. investigated the in vivo regulation of staphylococcal superantigen-like protein 1 [9]. They showed transcription of the *ssl1* gene increased when *S. aureus* was grown under nutrient deprived conditions that included early growth in murine abscesses. This represented the first time transcription of a staphylococcal superantigen-like gene was studied in vivo within *S. aureus* infecting animals.

A couple of papers dealt with *S. aureus* alpha-toxin. The Keogh et al. paper examined the role peptidyl-prolyl cis/trans isomerases (PPIases) have in regulating alpha-toxin and their contribution to *S. aureus* virulence in murine abscess and systemic models of infection [10]. A *ppiB* mutant that no longer encodes one of the PPIases produced less alpha-toxin and phenol-soluble modulins (PSMs) compared to the unmutated parent strain. A change in the host cell membrane that affected alpha-toxin activity was explored in the work done by Ziesemer et al. [11]. When sphingomyelinase was used to pre-treat airway epithelial cells, alpha-toxin heptamer formation was blocked, which led to a loss of transmembrane pore formation.

Two papers within this issue are centered on *S. aureus* enterotoxins. Grispoldi et al. studied enterotoxin production in *S. aureus* within canned meat [12]. The time between seaming and sterilization was examined. Enterotoxin was detected within one to two days depending on the incubation temperature of the canned meat. Heat treatment killed the *S. aureus*, but active enterotoxins could be present that would not be detected by serology. In the other enterotoxin paper, Fang et al. demonstrated that the application of purified staphylococcal enterotoxin C to murine mammary glands caused a significant increase in proinflammatory cytokines within those mammary glands compared to a phosphate buffered saline control [13]. Application of an anti-staphylococcal enterotoxin C antibody reduced both the inflammation and tissue damage within the mammary gland, strengthening the argument that staphylococcal enterotoxin C produced by *S. aureus* has an important role to play in mastitis development.

*S. aureus* is one of the leading causes of eye infection in humans. The role of toxins in *S. aureus* eye disease is well known. Astley et al.’s review paper describes the historic role of alpha-toxin in cornea damage [14]. Other hemolysins (i.e., delta-toxin and gamma toxin) and leukocidins have significantly lesser roles in regard to eye damage compared to the effect of alpha-toxin. The review notes that not much is known about the role of PSMs in corneal damage, a topic that needs further investigation.

Two papers within this special issue are focused on atopic dermatitis. Traisaeng et al. describes how butyric acid produced by one species of *Staphylococcus* was able to inhibit the growth of a *S. aureus* isolate derived from a patient with atopic dermatitis [15]. A derivative of the butyric acid was synthesized and led to a reduction in *S. aureus* mediated inflammation. The review by Seiti Yamada Yoshikawa et al. outlines the role of toxins in atopic dermatitis, a chronic skin disease that involves a significant inflammatory response [16]. They discuss how alpha toxin compromises E-cadherin integrity and interacts with sphinomyelin that in turn leads to lysis of keratinocytes. Newer studies they present suggest that staphylococcal toxins not only promote inflammation, but they may also serve as a counterbalance to some of the host regulatory mechanisms.

The paper by Habib et al. used a bioinformatic approach to find toxin–antitoxin systems in *S. aureus* [17]. They found 39% of the toxin–antitoxin systems are within the seven *S. aureus* pathogenicity islands. Furthermore, a new *S. aureus* toxin–antitoxin system was identified where the antitoxin is a transcriptional autoregulator and the toxin inhibits the autoregulation.

Papers by Kailasan et al. [18] and Ouyang et al. [19] explored treatment options for *S. aureus* infections. Kailasan et al. generated a library of leukotoxin gene mutations that targeted functional domains of the leukocidin protein that were in turn used to make polyclonal antibodies to the lead toxoid candidate. A combination of antibodies to various toxins that included the new anti-leukocidin antibody completely neutralized the cytotoxic properties of the *S. aureus*. On the other hand, Ouyang et al. tested a bibenzyl compound for its anti-virulence capabilities against *S. aureus*. Mice treated with the drug had a significantly higher survival rate than untreated mice.

Lastly, the paper by Tuchscherr et al. examined several strains of *S. aureus* for their genotype and virulence capabilities [20]. These strains were split into low-cytotoxicity and high-cytotoxicity arms for use in two murine infection models. Both groups persisted within the mice and were able to cause infections. The low-cytotoxicity strains grew to higher numbers and were not cleared to the same extent as the high-cytotoxicity strains, suggesting this adaptation could be important in the development of chronic infections.
